# Lead Exposure Is Associated with Decreased Serum Paraoxonase 1 (PON1) Activity and Genotypes

**DOI:** 10.1289/ehp.9163

**Published:** 2006-05-18

**Authors:** Wan-Fen Li, Mei-Hung Pan, Meng-Chu Chung, Chi-Kung Ho, Hung-Yi Chuang

**Affiliations:** 1 Division of Environmental Health and Occupational Medicine, National Health Research Institutes, Zhunan, Taiwan; 2 Graduate Institute of Public Health, College of Health Sciences, Kaohsiung Medical University, Kaohsiung, Taiwan; 3 Department of Occupational Medicine, Kaohsiung Medical University Hospital, Kaohsiung, Taiwan

**Keywords:** atherosclerosis, lead, metal, paraoxonase, polymorphism

## Abstract

Lead exposure causes cardiac and vascular damage in experimental animals. However, there is considerable debate regarding the causal relationship between lead exposure and cardiovascular dysfunction in humans. Paraoxonase 1 (PON1), a high-density lipoprotein-associated antioxidant enzyme, is capable of hydrolyzing oxidized lipids and thus protects against atherosclerosis. Previous studies have shown that lead and several other metal ions are able to inhibit PON1 activity *in vitro*. To investigate whether lead exposure has influence on serum PON1 activity, we conducted a cross-sectional study of workers from a lead battery manufactory and lead recycling plant. Blood samples were analyzed for whole-blood lead levels, serum PON1 activity, and three common *PON1* polymorphisms (Q192R, L55M, −108C/T). The mean blood lead level (± SD) of this cohort was 27.1 ± 15 μg/dL. Multiple linear regression analysis showed that blood lead levels were significantly associated with decreased serum PON1 activity (*p* < 0.001) in lead workers. This negative correlation was more evident for workers who carry the R192 allele, which has been suggested to be a risk factor for coronary heart disease. Taken together, our results suggest that the decrease in serum PON1 activity due to lead exposure may render individuals more susceptible to atherosclerosis, particularly subjects who are homozygous for the R192 allele.

Although leaded gasoline has been phased out for decades, lead continues to be a public health concern. Lead accumulates in the human body and has been linked to increased cancer and cardiovascular mortality (Lustberg and Silbergeld 2002). Most studies of the cardiovascular effects of lead in humans have focused on lead’s causal relationship with hypertension. Although the results are controversial, a meta-analysis has revealed a weak but significantly positive association between blood lead level and blood pressure (Nawrot et al. 2002). Meanwhile, an association between lead exposure and serum cholesterol and lipoprotein levels was found in workers of battery and recycling factories (el-Gazzar et al. 1989; Kristal-Boneh et al. 1999), indicating a risk for the development of atherosclerosis. Studies in animals also suggested that lead exposure may promote atherosclerosis, as shown by fatty degeneration and sclerotic changes on artery walls of lead-intoxicated rats (Skoczynska et al. 1993). A recent study in the general U.S. population has shown an association of blood lead with elevated prevalence of peripheral arterial disease, a disorder characterized by atherosclerosis in the arteries of the lower extremities (Navas-Acien et al. 2004).

Human paraoxonase 1 (PON1) is a serum esterase transported on high-density lipoprotein (HDL) particles. PON1 is thought to attenuate the oxidation of low-density lipoprotein (LDL) and therefore protect against the development of atherosclerosis, although the exact mechanisms and substrates for PON1 are unclear. Animal studies have strongly supported the protective role of PON1 in atherosclerosis. *PON1*-knockout mice were prone to develop atherosclerotic plaques when fed a high-fat diet (Shih et al. 1998), and these animals had increased oxidative stress in both serum and macrophages (Rozenberg et al. 2003). On the other hand, *PON1*-overexpressing mice showed a reduction in atherosclerotic lesion formation (Tward et al. 2002), and their HDL was more resistant to oxidative damage (Oda et al. 2002). The role of PON1 in cardiovascular disease has also been suggested by epidemiologic studies. A coding region polymorphism (Q192R) of the human *PON1* gene (Wheeler et al. 2004) and low serum PON1 activity levels (Jarvik et al. 2000; Mackness et al. 2001, 2003) were both associated with increased incidence of coronary heart disease.

The enzymatic activity of PON1 is mainly determined by the Q192R polymorphism (Humbert et al. 1993). However, a variety of environmental and pharmaceutical factors are able to modulate PON1 activity as well. Previous studies have shown that various metals, including lead at concentrations < 1 μM, caused significant inhibition of PON1 activity *in vitro* (Cole et al. 2002; Debord et al. 2003). Whether long-term, low-level lead exposure has any effect on PON1 activity *in vivo* is yet to be investigated.

The aim of the present study was to understand whether lead exposure has any effects on serum PON1 activity and serum cholesterol levels. We conducted a cross-sectional study to evaluate the relationship between blood lead level, serum cholesterol and lipoprotein levels, and serum PON1 activity in a cohort of workers of lead-acid battery and recycling plants. Results of this study may help us understand the possible interaction between lead and polymorphisms of genes that encode proteins known to be involved in regulation of atherosclerosis.

## Materials and Methods

### Study population

We carried out this study in a lead-acid battery manufactory and a lead recycling plant, where workers have been followed since 1990 with annual health examinations, including physical examination, blood lead test, hematology test, serum lipids test, and liver and renal function tests. A total of 597 workers 21–61 years of age (mean ± SD, 40.2 ± 15.3 years) were enrolled in this study. We collected blood samples on-site during the annual health examination in 2002. Buffy coat isolated from EDTA-treated blood was used for genomic DNA preparation, whereas serum was collected for the PON1 activity assay. All samples were stored at −20°C until measurement. This protocol was approved by the institutional review board of Kaohsiung Medical University, and informed consent was obtained from subjects before the study.

### Chemicals and materials

Phenyl acetate (purity 99%) was purchased from Sigma-Aldrich (St. Louis, MO, USA), and paraoxon (purity 98.5%) and diazinon-oxon (purity 97.4%) were obtained from Chem Service (West Chester, PA, USA). Ultraviolet-transparent 96-well plates were purchased from Costar (Cambridge, MA, USA), and standard 96-well plates were from Nunc (Roskilde, Denmark).

### Blood lead level measurement

Blood lead levels were analyzed by a Zeeman effect graphite furnace atomic absorption spectrometer (PerkinElmer 5100 PC with AS 60 auto-sampler; PerkinElmer, Wellesley, MA, USA).

### PON1 activity assay

PON1 arylesterase activity was measured in 9 mM Tris-HCl, pH 8, and 0.9 mM calcium chloride with 3.26 mM phenyl acetate at 27°C (Furlong et al. 1988). The rate of phenol generation was monitored at 270 nM, and a molar extinction coefficient of 1,310 was used to calculate the enzyme activity. PON1 paraoxonase activity was measured using 1.2 mM paraoxon in 0.1 M Tris-HCl, pH 8.5, 2 mM CaCl_2_, and 2 M NaCl at 37°C (Furlong et al. 1988). Reaction was monitored at 405 nM, and an extinction coefficient of 18 mM^−1^cm^−1^ was used for activity calculation. PON1 activity for hydrolyzing diazoxon was determined as previously described (Furlong et al. 1989; Richter and Furlong 1999), with minor modification. PON1 diazoxonase activity was measured using 1 mM diazoxon in 0.1 M Tris-HCl, pH 8.5, 2 mM CaCl_2_, and 2.5 M NaCl at 27°C. Reaction was monitored at 270 nM, and an extinction coefficient of 3 mM^−1^cm^−1^ was used for calculation. PON1 activity was expressed as micromoles of hydrolysis product formed per minute per liter or milliliter. The assays were performed in a 96-well microplate spectrophotometer SPECTRAMax 190 (Molecular Devices, Sunnyvale, CA, USA).

### PON1 *genotyping*

Genomic DNA was extracted from buffy coat using a commercial kit (QIAamp DNA Mini Kit; Qiagen, Hilden, Germany). All genotyping was conducted by polymerase chain reaction amplification followed by polymorphism-specific restriction enzyme digestion and gel analysis. The Q192R and L55M polymorphisms were determined following a protocol developed by Humbert et al. (1993), whereas the promoter region polymorphism −108C/T was determined according to Brophy et al. (2001).

### Statistical analysis

Differences between groups were analyzed using one-way analysis of variance (ANOVA). The magnitude of the correlation between PON1 activity, blood lead level, and serum lipids was assessed by Pearson coefficient of correlation. Deviation from Hardy-Weinberg equilibrium was evaluated using chi-square tests. Multiple linear regression analysis, controlling for *PON1* genotypes and potential confounding factors, was used to test the association between PON1 activities, or serum lipids, and blood lead level.

## Results

A total of 597 workers were evaluated for their blood lead levels, serum lipids, and PON1 activities. The mean (± SD) blood lead level of this cohort was 27.1 ± 15 μg/dL. Workers were divided into low (≤ 10 μg/dL), medium (10–40 μg/dL), and high (> 40 μg/dL) exposure groups based on their blood lead levels (Table 1), where 10 μg/dL is the criterion for elevated blood levels in children and pregnant women set by the U.S. Centers for Disease Control and Prevention (CDC 2005) and 40 μg/dL is the highest level accepted by the standards of the U.S. Occupational Safety and Health Administration (OSHA 2003) and the Taiwan government (Institute of Occupational Safety and Health 2002). The high-exposure group included mostly males with the longest work history and the highest smoking rate. No difference was found in systolic or diastolic blood pressure. Only triglyceride level was different among the three groups, with the medium-exposure group having the highest level of triglycerides. Multiple linear regression analysis revealed that, after controlling for age, sex, work history, smoking, and body mass index (BMI), blood lead was negatively associated with triglycerides but positively associated with HDL cholesterol (Table 2).

Interestingly, we found significant differences in serum PON1 activities among the three exposure groups (Table 1). We determined PON1 activities using three different substrates, phenyl acetate (arylesterase), paraoxon, and diazoxon, and all three activities were decreased with increasing exposure level. The average paraoxonase activity of the high-exposure group was approximately 18% lower than the low-exposure group (714.4 ± 343.5 vs. 869.2 ± 399.9 μmol/min/L).

We determined three common polymorphisms of the human *PON1* gene, and the gene frequencies of Q192R, L55M, and −108C/T were similar to those reported in the literature for the Chinese population (Wang et al. 2003) (Table 3). As expected, paraoxonase and diazoxonase activities were influenced by the Q192R and −108C/T polymorphisms. Our data showed that arylesterase activity was also affected by the Q192R polymorphism, which is in contrast to the general belief that arylesterase activity is independent of the polymorphism at position 192.

**Figure 1:**
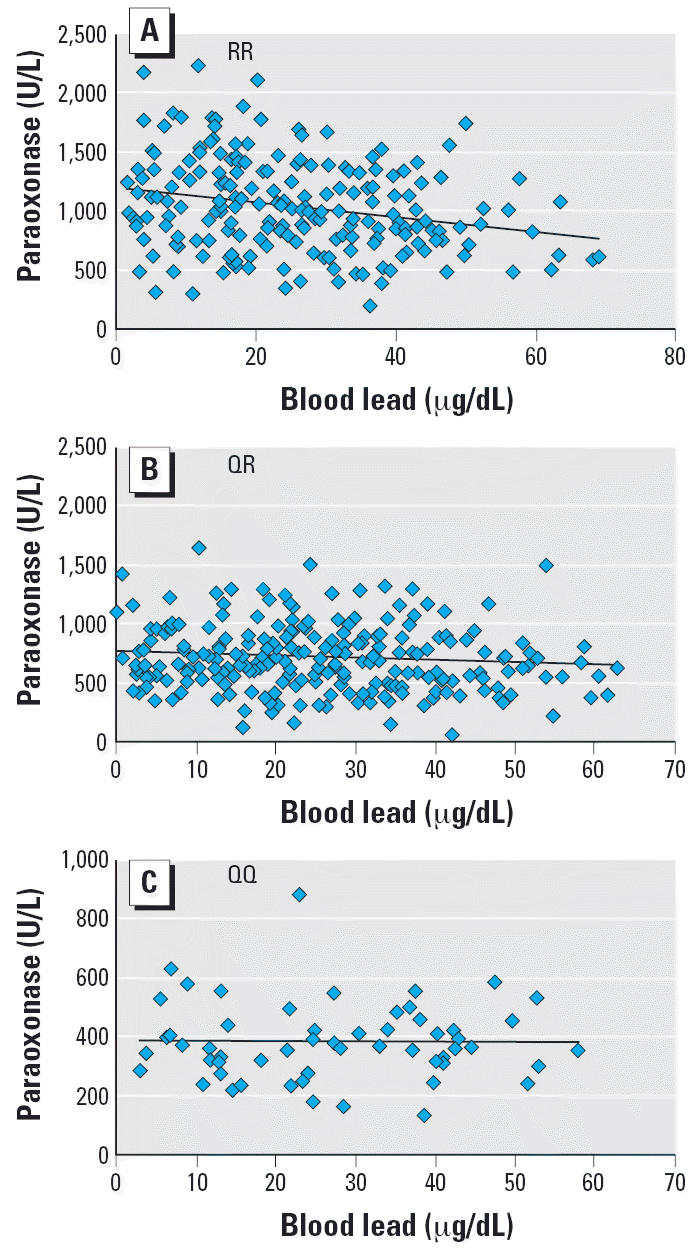
Linear regression trend between blood lead level and serum paraoxonase activity among lead workers in Taiwan shown by *PON1* Q192R genotype. (*A*) RR homozygotes (*r* = −0.251, *p* < 0.001). (*B*) QR heterozygotes (*r* = −0.101, *p* < 0.122). (*C*) QQ homozygotes (*r* = −0.007, *p* < 0.959).

Table 4 represents a multivariate linear regression model for serum PON1 activities. *PON1* polymorphisms, blood lead, HDL cholesterol, sex, smoking, age, and work history were included in the model. Collectively, these variables were associated with 35.3% of variance in arylesterase and paraoxonase activities and 64.9% of variance in diazoxonase activity. Blood lead was found to be an independent factor affecting serum PON1 activity. An increase of 1 μg/dL in blood lead would result in a decrease of 0.403 μmol/min/mL in arylesterase activity, 4.059 μmol/min/L in paraoxonase activity, and 29.244 μmol/min/L in diazoxonase activity. When subjects were separated by their Q192R genotype (Figure 1), the negative correlation between blood lead and paraoxonase activity was significant only in subjects of RR genotype (*r* = −0.251, *p* < 0.001), but not in subjects of QR (*r* = −0.101, *p* = 0.122) or QQ genotype (*r* = −0.007, *p* = 0.959). This result indicates significant effects on serum paraoxonase activity by interaction between blood lead and the Q192R polymorphism.

## Discussion

The main finding of the present study is that lead exposure is associated with decreased serum PON1 activity. The reverse dose–response relationship between blood lead and PON1 activity was demonstrated in a large cohort of active lead workers (*n* = 597). Moreover, our study is the first report in humans showing an inhibitory effect of heavy metal exposure on PON1 activity. This is consistent with the results of previous *in vitro* studies in which various metals, including lead, have been shown to inhibit the activity of purified human PON1 (Debord et al. 2003). In the study by Cole et al. (2002), lead chloride at < 1 μM was able to inhibit the arylesterase activity of purified human PON1 by > 50%. The average blood lead of our cohort was 27 μg/dL, equal to 1.3 μM, which is comparable to the doses tested by Cole et al. (2002). This indicates that our finding is biologically plausible, rather than merely a statistical coincidence.

Our results also show a weak effect of lead exposure on serum lipids, where a negative association was found between blood lead and triglycerides, and a positive association was found for HDL cholesterol. It agrees with a previous report in which HDL cholesterol was higher among lead workers than in controls (Kristal-Boneh et al. 1999). Although increased HDL cholesterol and low triglyceride levels could be argued to be a “protective effect,” we found that it may not be the case. First, the effect of lead exposure on serum lipids was very weak (β = 0.066 for HDL; β = −0.001 for log triglycerides) with marginal significance (*p* = 0.043 for HDL; *p* = 0.041 for log triglycerides) (Table 2). It is unlikely that this effect on serum lipids would result in any beneficial outcome. Second, the antioxidant function of HDL particles is mainly attributed to PON1. Because PON1 activity is decreased, the protective effect of HDL is likely to be damaged, as well. Therefore, even though HDL cholesterol is slightly increased with blood lead level, its protection against atherosclerosis may not be increased.

The mechanism by which heavy metals inhibit serum PON1 activity is still not clear. Gonzalvo et al. (1997) suggested that metal ions, such as copper and mercury, bind to the free sulfhydryl group of the enzyme. PON1 has three cysteine residues; two of them form a disulfide bond, and the third one—located at residue 284—is free. Although this residue (Cys284) is not at the active site for hydrolytic activity of PON1, its mutation or blockage is likely to destabilize the structure of PON1 and affect its function (Harel et al. 2004). More important, this free sulfhydryl group is required for protection of PON1 against LDL oxidation (Aviram et al. 1998). If lead, acting like other divalent metal ions, binds to the free sulfhydryl group of PON1, it will reduce not only the hydrolytic activity of PON1 but also its antioxidant function.

Lead is well known to cause cardiovascular damage, including atherosclerosis. PON1 plays an important role in protection against this disease by removing LDL peroxides, whose accumulation is a critical step in the development of atherosclerosis. It seems reasonable to assume that the decreased PON1 activity found among lead workers also represents a reduced protection against LDL oxidation, thereby increasing the accumulation of lipid peroxides and, eventually, promoting atherosclerosis. The relationship between blood lead and other antioxidant enzymes has been reported previously by Ito et al. (1985), who found that occupational lead exposure was associated with decreased superoxide dismutase activity while the levels of serum lipid peroxides were increased. Our data provide further evidence that lead exposure may increase oxidative stress by inhibiting anti-oxidant enzymes.

Interestingly, the inhibitory effect of lead on PON1 activity is influenced by the Q192R polymorphism. The present study indicates that, in terms of paraoxonase activity, subjects who are homozygous for the R allele are more susceptible to lead toxicity than are subjects of other genotypes (Figure 1). The lower stability of the R allele compared to the Q allele was also observed in an oxidizing environment. The HDL isolated from the RR subjects retained < 1% of antioxidant function after 6 hr of incubation, whereas the QQ HDL kept > 50% of its original activity (Mackness et al. 1998). On the other hand, the R allele was also more sensitive to the beneficial effects of environmental factors, because the effects of an antiatherogenic diet (Bub et al. 2002; Tomas et al. 2001) or physical activity (Senti et al. 2000) were found only in subjects carrying the R allele. Together with our data, all the findings suggest a significant interaction between the Q192R polymorphism and environmental factors. This implies that the effect of the Q192R polymorphism on a particular trait, such as cardiovascular disease, may be enhanced or diluted by certain environmental factors.

In summary, lead exposure is associated with decreased serum PON1 activity, and this inhibitory effect is most obvious for subjects who carry two R alleles. Whether this event leads to more profound cardiovascular damage in lead workers is yet to be explored. However, because of the protective role of PON1 in the development of atherosclerosis, serum PON1 activity could be used as a bio-marker to monitor the cardiovascular health among lead workers.

## Figures and Tables

**Table 1:** Demographic characteristics of lead workers in Taiwan.

	BPb (μg/dL)		
Characteristic	Low (BPb ≤ 10)	Medium (10 < BPb ≤ 40)	High (BPb > 40)
No.	85	384	128
Age (year)	37.5 ± 8.5	39.7 ± 8.7*	40.7 ± 8.3*
Sex (% male)	65.9	72.7*	85.2*
Smoking (%)	16.5	37.4*	50.0*
Years working	9.1 ± 6.9	10.7 ± 5.8*	10.9 ± 5.4*
BMI (kg/m^2^)	23.3 ± 3.5	24.5 ± 3.7*	23.9 ± 3.8
SBP (mmHg)	126.5 ± 12.7	127.3 ± 13.7	128.2 ± 14.7
DBP (mmHg)	77.5 ± 8.9	78.5 ± 10.4	76.7 ± 10.9
Triglycerides (mg/dL)	125.9 ± 76.9	151.6 ± 131.3*	124.0 ± 73.8
Total cholesterol (mg/dL)	187.1 ± 29.3	195.3 ± 34.6	191.7 ± 37.5
HDL (mg/dL)	49.0 ± 12.1	48.8 ± 11.6	49.1 ± 10.7
Arylesterase (μmol/min/mL)	103.9 ± 33.5	93.6 ± 32.3*	90.9 ± 35.8*
Paraoxonase (μmol/min/L)	869.2 ± 399.9	826.8 ± 394.1*	714.4 ± 343.5*
Diazoxonase (μmol/min/L)	7,001 ± 3,894	6,341 ± 3,581	6,164 ± 3,677

Abbreviations: BPb, blood lead level; DBP, diastolic blood pressure; SBP, systolic blood pressure. Data are mean ± SD.

* *p* < 0.05 by one-way ANOVA or chi-square test.

**Table 2:** Multiple linear regression analysis for association of blood lead level with serum lipids.

	HDL cholesterol		Triglycerides^a^		Total cholesterol	
Variable	β (SE)	*p*-Value	β (SE)	*p*-Value	β (SE)	*p*-Value
Blood lead	0.066 (0.033)	0.043	−0.0014 (0.001)	0.041	−0.122 (0.104)	0.238
Arylesterase	0.053 (0.014)	0.000	−0.00003 (0.000)	0.912	0.0651 (0.044)	0.138
Sex	−7.367 (1.157)	0.000	0.0617 (0.024)	0.011	−0.903 (3.659)	0.805
Smoking	−1.952 (1.106)	0.078	0.0582 (0.023)	0.012	−4.362 (3.496)	0.213
Age	0.00071 (0.055)	0.990	0.0015 (0.001)	0.187	0.570 (0.175)	0.001
Work history^b^	−5.625 (3.902)	0.150	0.107 (0.081)	0.189	2.849 (12.3)	0.818
BMI	−0.749 (0.124)	0.000	0.0254 (0.003)	0.000	2.317 (0.393)	0.000
Intercept	67.960 (3.838)	0.000	1.335 (0.080)	0.000	112.188 (12.1)	0.000
*R*^2^	0.217		0.225		0.102	

a Triglycerides values were log-transformed to improve normality.

b Work history represents the ratio of work years to age.

**Table 3:** Serum PON1 activities and *PON1* genotypes in lead workers.

*PON1* genotype	Arylesterase (μmol/min/mL)	Paraoxonase (μmol/min/L)	Diazoxonase (μmol/min/L)
Q192R			
QQ (*n* = 61)	124.5 ± 30.9	382.9 ± 130.5	11,113 ± 2,797
QR (*n* = 256)	102.6 ± 30.0	723.3 ± 294.2	7,893 ± 2,768
RR (*n* = 210)	76.5 ± 27.5	1035.5 ± 391.3	3,223 ± 1,594
*p*-Value	< 0.001	< 0.001	< 0.001
−108C/T			
CC (*n* = 154)	112.0 ± 33.8	696.7 ± 379.4	8,693 ± 3,560
CT (*n* = 265)	93.0 ± 30.4	826.3 ± 396.0	6,206 ± 3,334
TT (*n* = 108)	74.2 ± 26.0	935.4 ± 335.2	3,629 ± 2,113
*p*-Value	< 0.001	< 0.001	< 0.001
L55M			
LL (*n* = 497)	95.3 ± 33.5	831.2 ± 387.0	6,438 ± 3,698
LM (*n* = 30)	84.5 ± 28.1	507.1 ± 251.6	5,856 ± 2,666
*p*-Value	< 0.001	< 0.001	< 0.001

Allele frequencies for polymorphisms are as follows: Q192R, Q = 0.359, R = 0.641; −108C/T, C = 0.544, T = 0.456; and L55M, L = 0.972, M = 0.028. No individuals were homozygous for the M allele in this cohort. Data are mean ± SD. Statistical significance between genotypes was analyzed by one-way ANOVA and Scheffe test.

**Table 4:** Multivariate regression model for associations with serum arylesterase, paraoxonase and diazoxonase activities.

	Arylesterase			Paraoxonase			Diazoxonase		
	β	SE	*p*-Value	β	SE	*p*-Value	β	SE	*p*-Value
Q192R									
QR vs. RR	21.611	2.923	< 0.001	−346.633	35.251	< 0.001	4489.059	754.545	< 0.001
QQ vs. RR	41.627	4.671	< 0.001	−686.910	57.074	< 0.001	7585.392	376.229	< 0.001
−108C/T									
CT vs. CC	−9.854	3.077	0.001	−54.361	37.322	0.146	−762.829	247.806	0.002
TT vs. CC	−19.713	4.418	< 0.001	−124.209	50.611	0.014	−1366.551	334.128	< 0.001
55L/M									
LM vs. LL	−25.393	5.331	< 0.001	−150.240	62.424	0.016	−3062.718	429.351	< 0.001
Blood lead	−0.403	0.086	< 0.001	−4.059	1.026	< 0.001	−29.244	6.932	< 0.001
HDL cholesterol	0.411	0.113	< 0.001	3.514	1.348	0.009	20.441	9.086	0.025
Sex	2.211	3.179	0.487	30.724	38.245	0.422	−13.215	256.047	0.959
Smoking	3.336	2.922	0.254	7.450	35.265	0.833	167.126	235.341	0.478
Age	−0.090	0.145	0.535	1.243	1.758	0.480	−11.638	11.662	0.319
Work history	−14.209	10.288	0.168	−223.850	122.519	0.068	−1402.009	828.617	0.091
Intercept	84.146	9.368	< 0.001	1031.287	113.345	< 0.001	4683.922	754.545	< 0.001
*R*^2^		0.353			0.353			0.649	
